# Cationic Vitamin E-TPGS Mixed Micelles of Berberine to Neutralize Doxorubicin-Induced Cardiotoxicity via Amelioration of Mitochondrial Dysfunction and Impeding Apoptosis

**DOI:** 10.3390/molecules29051155

**Published:** 2024-03-05

**Authors:** Abdelkader A. Metwally, Samayita Ganguly, Nora Biomi, Mingyi Yao, Tamer Elbayoumi

**Affiliations:** 1 Department of Pharmaceutics, College of Pharmacy, Health Science Center (HSC), Kuwait University, P.O. Box 24923, Safat 13110, Kuwait; abdelkader.metwally@ku.edu.kw; 2 Department of Pharmaceutics and Industrial Pharmacy, Faculty of Pharmacy, Ain Shams University, Abbasseya, Cairo 11566, Egypt; 3 Parkinson’s Disease Research Unit, Department of Neurobiology, Barrow Neurological Institute, Dignity Health/St. Joseph’s Hospital and Medical Center, 350 W. Thomas Rd., Phoenix, AZ 85013, USA; samayita.ganguly@barrowneuro.org; 4 Pharmacology and Toxicology Program, New College of Interdisciplinary Arts and Sciences, West Valley Campus, Arizona State University, N. 47th Ave & University Way, Glendale, AZ 85306, USA; nbiomi@asu.edu; 5 Department of Pharmaceutical Sciences, Glendale Campus (CPG), College of Pharmacy, Midwestern University, 218-Cholla Hall, 19555 N. 59th Ave., Glendale, AZ 85308, USA; 6 College of Graduate Studies, Midwestern University, Dr. Arthur G. Dobbelaere Science Hall 350D, 19555 N. 59th Ave., Glendale, AZ 85308, USA

**Keywords:** anthracycline antibiotics, doxorubicin, cardiomyopathy, apoptosis, berberine, mitohormetic, mitochondrial membrane potential, D-α-tocopheryl polyethylene glycol succinate, mixed micelles, cytoprotection

## Abstract

Anthracycline antibiotics, namely, doxorubicin (DOX) and daunorubicin, are among the most widely used anticancer therapies, yet are notoriously associated with severe myocardial damage due to oxidative stress and mitochondrial damage. Studies have indicated the strong pharmacological properties of Berberine (Brb) alkaloid, predominantly mediated via mitochondrial functions and nuclear networks. Despite the recent emphasis on Brb in clinical cardioprotective studies, pharmaceutical limitations hamper its clinical use. A nanoformulation for Brb was developed (mMic), incorporating a cationic lipid, oleylamine (OA), into the TPGS-mixed corona of PEGylated-phosphatidylethanolamine (PEG-PE) micelles. Cationic TPGS/PEG-PE mMic with superior Brb loading and stability markedly enhanced both intracellular and mitochondria-tropic Brb activities in cardiovascular muscle cells. Sub-lethal doses of Brb via cationic OA/TPGS mMic, as a DOX co-treatment, resulted in significant mitochondrial apoptosis suppression. In combination with an intense DOX challenge (up to ~50 µM), mitochondria-protective Brb-OA/TPGS mMic showed a significant 24 h recovery of cell viability (*p* ≤ 0.05–0.01). Mechanistically, the significant relative reduction in apoptotic caspase-9 and elevation of antiapoptotic Bcl-2 seem to mediate the cardioprotective role of Brb-OA/TPGS mMic against DOX. Our report aims to demonstrate the great potential of cationic OA/TPGS-mMic to selectively enhance the protective mitohormetic effect of Brb to mitigate DOX cardiotoxicity.

## 1. Introduction

Chemotherapy-induced cardiotoxicity imposes substantial restrictions on drug development, as well as in the clinical management of existing anti-neoplastic agents. Anthracycline-based chemotherapy, specifically doxorubicin and daunorubicin, can result in the development of cumulative and progressively developing cardiomyopathy. Specifically, doxorubicin (DOX) is one of the most highly prescribed chemotherapeutic agents thanks to its broad spectrum of efficacy against hematological malignancies and solid tumors [1,2]. The clinical effectiveness of DOX is often compromised due to the development of cumulative dose-dependent cardiac toxicity, manifested as arrhythmia, irreversible dilated cardiomyopathy, left ventricular dysfunction, and congestive heart failure [3,4]. Early-onset and progressive anthracycline cardiotoxicity, following a course of therapy, can occur within a year or may not become apparent until years after the cessation of treatment. The most difficult to predict and manage clinically is the development of cardiomyopathy and, ultimately, congestive heart failure, which depends on the cumulative lifetime dose. For this reason, a total cumulative dose of 550 mg/m^2^ is considered to be the therapeutic endpoint of doxorubicin therapy [2,5].

Since DOX accumulates primarily in nuclei and mitochondria, usually abundant in cardiomyocytes, this mainly explains the cardio-selective toxicity of the drug. Combined with a relatively smaller active antioxidant network in the heart, damage-associated molecular patterns involving mitochondria in cardiomyocytes or vascular cells are considered at the top of the list. DOX has been shown to inflect immediate and direct damage on the mitochondrial respiration system through redox cycling and oxidative-stress-mediated mitochondrial uncoupling early on. Furthermore, direct interference with mitochondrial proteins and structures involves detrimental DOX-cardiolipin complex formation and the dose-dependent opening of the mitochondria permeability transition pores (mPTPs), causing calcium loss and cytochrome c release into the cytoplasm. In fact, DOX-induced oxidative stress and induction of the cyclosporin A-sensitive mPTP lead to the rupture of the mitochondrial outer membrane—due to the accumulation of proapoptotic proteins, such as Bax, and reduction in anti-apoptotic proteins (e.g., Bcl-2)—and release proapoptotic factors, with the ultimate activation of the intrinsic apoptotic pathway, and these have been the most extensively described mechanisms for the loss of cardiomyocytes [6,7,8,9]. Moreover, nuclear-mediated DOX cardiotoxic cascade, starting with the inhibition of topoisomerase2β, results in nuclear damage, p53 activation, and downstream inhibition of mitochondrial function. Such mito-nuclear interplay, consequential remodeling of cardiomyocyte metabolic flux and mitochondrial biogenesis, and autophagy flux alterations collectively underlie the broad DOX-induced cardiomyocyte loss. Therefore, mitochondrial stability and protection have emerged as the primary factors for both the extent of the molecular and structural damage caused by DOX and the cardiovascular resilience against it.

Several natural compounds, such as isoquinoline alkaloids derived from plants of the Berberidaceae family, have attracted attention, owing to their preferential mitochondrial accumulation and activities. Specifically, many pharmacological studies have demonstrated strong anti-inflammatory, antidiabetic, and antiarrhythmic effects of Berberine (Brb) and anti-proliferative and antiangiogenic properties, primarily mediated through mitochondrial functions and networks [5,8,9,10,11,12,13]. This is mainly explained through the selective accumulation of the monovalent cation of Brb within mitochondria as the most negatively charged cellular organelle (Δψ about −180 mV). In fact, the membrane-potential-driven uptake of Brb alkaloid by mitochondria in living mammalian cells can reach up to 1000 times higher than in cytosol [10,14,15].

Therefore, in recent reports, the mitohormetic response of Brb has been strongly implicated in its proposed clinical cytoprotective properties, namely those that are cardioprotective [5,9,16]. Sublethal mitochondrial stress from mitochondria-targeted drugs, such as Brb, has demonstrated beneficial effects on cells and organisms against larger subsequent stress-induced damages or death [17,18]. Mito-nuclear communications underlying mitohomesis have been shown to be involved in diverse cytosolic and nuclear signaling pathways, including ROS, the AMPK pathway, and the mitochondrial unfolded protein response (UPRmt) [7,9,10]. In this regard, sub-lethal Brb would induce mitochondrial stress protection against DOX-stress-induced myocardial cell damage through multiple mechanisms, such as the mitochondrial respiratory-chain-mediated ROS production/redox pathway, AMP/ATP-induced AMPK signaling pathway, NAD+/NADH-mediated sirtuins (SIRT1-3) pathway, and UPRmt pathway [5,6,8,16,19]. Essentially, adaptive responses to low-dose Brb-mediated mitochondrial energy stress influence the signal interactions downstream of these pathways and their crosstalk with the mito-nuclear communication pathways implicated in broad DOX-induced cardiomyocyte loss. The effective administration of Brb would ultimately enhance the adaptiveness of cardiomyocytes to DOX-induced myocardial apoptosis and cardiac dysfunction by upregulating the transcription involved in resolving the antioxidant response, metabolic adaptation, and cell survival [5,7,8,10,19].

Pharmaceutically, the alkaloid Brb, as a hydrophilic compound, is considered to be a class III drug, characterized by limited aqueous solubility, except in basic solution, and an extremely low permeability [20]. Under physiological conditions, it mainly exists in a positively charged protonated form and, thus, cannot sufficiently diffuse through cell membranes. Yet, once inside the cell, Brb cations accumulate primarily in energized mitochondria, then move to the nucleus and other organelles, following high saturation in the mitochondria [11,15,21].

Given the valuable mitohormetic effect of Brb related to low dosage, this mitochondria-targeted drug has been extensively pursued for myocardial protection against IRI and DOX toxicity, and recently with dialectic clinical application [5,8,12,22]. Unfortunately, preclinical data have also indicated a very poor oral bioavailability (≤0.35) of Brb due to its poor solubility, combined with low gastrointestinal absorption (partially attributed to being a substrate of intestinal P-gp transporters), extensive first-pass metabolism, and rapid systemic elimination. Most importantly, Brb is also notorious for its relatively short plasma half-life (T_1/2_ is about 30–45 min), which represents a clinical challenge, even after intravenous administration of a simple drug solution [14,15,20,21,22,23].

**Figure 1:**
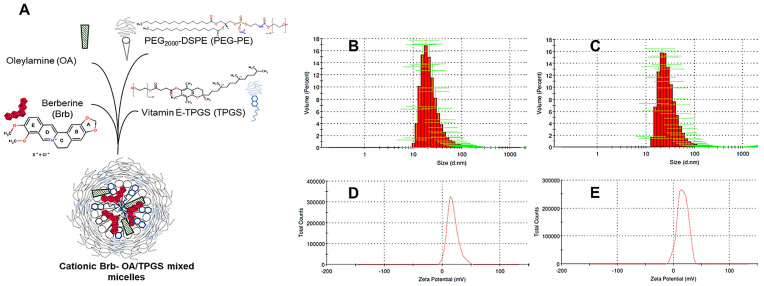
A diagram of cationic vitamin E-TPGS mixed berberine micelles (Brb) illustrates the chemical structures of cationic mMic components (**A**) and the characteristics of empty vs. Brb-loaded cationic mMic formulations, in terms of particle size distribution (in nm, panels (**B**) vs. (**C**)), and average measured surface charge, ζ (in mV, panels (**D**) vs. (**E**)), respectively. (*n* = 3–4, mean ± SD, *p* ≤ 0.05).

Earlier, a mixed micelle (mMic) formulation of Brb was investigated by our lab to enhance the solubilization efficiency of PEG-phospholipid micellar carriers [14,24,25] by including D-α-tocopheryl polyethylene glycol 1000 succinate (TPGS) as an additional component—a PEGylated derivative of natural Vitamin E (VE, α-tocopherol), commonly utilized as a pharmaceutical solubilizer, absorption enhancer, and vehicle for lipidic drug formulations [14,25,26]. Such a mixed micellar system did not only improve the solubilization capacity of Brb due to the increased core volume of mixed micelles created by the planar chromane moiety of VE, but also provided better protection for the active isoquinolone structure of Brb (Figure 1A), effectively enhancing both Brb encapsulation and stability [14,25]. Our previous data and other reports have demonstrated the pharmaceutical advantages of such a TPGS-based mMic platform as a delivery system for Brb [14,27,28].

Here, a combined mixed micellar system was developed via the additional incorporation of a cationic lipid component (oleylamine, OA) along with the TPGS-mixed coronal matrix of the PEGylated-phosphatidylethanolamine (PEG-PE) micelles (Figure 1A). Such cationic TPGS/PEG-PE mMic nanocarriers of Brb would demonstrate that they offer not only superior drug loading and stability under physiological conditions, but can also enhance both the intracellular and mitochondria-tropic delivery of Brb molecules [5,7,8,29]. The mitochondria-targeted accumulation of a sub-lethal dose of Brb via this cationic OA/TPGS mMic system would offer significant mitochondrial apoptosis suppression and even the recovery of myocardial cell viability and energetics following the acute oxidative and non-oxidative toxicities incurred by elevated doses of DOX. Our current report aims to demonstrate the great potential of cationic OA/TPGS-mMic to selectively enhance the protective mitohormetic effect of Brb for the effective mitigation of DOX cardiotoxicity.

## 2. Results

The mitochondria-tropic accumulation of Brb has been proposed as a critical molecular prerequisite of the therapeutic action of this drug for preventing myocardial toxicity induced by DOX anticancer chemotherapy. Hence, cationic TPGS/PEG-PE mixed micelles (mMic) were developed for the enhanced intracellular delivery of Brb to target its accumulation in impaired mitochondria in cardiomyocytes following toxic exposure to DOX. The targeted OA/TPGs-mMic nanoformulation of Brb was then investigated to improve its cardioprotective effects against the myocardial toxicity of DOX via attenuating mitochondrial dysfunction and apoptosis.

### 2.1. Nanoformulation of Cationic Brb–Mixed Micelles and Physico-Chemical Characterization

In aqueous HEPES-buffer saline, the original anionic micelles and the cationic micelles could self-assemble upon the hydration of the lipid film with the buffer solution. Figure 1A illustrates the chemical structures of the different components of the prototype cationic micelles components. The monomodal size distribution of the empty and Brb-loaded cationic micelles is shown in Figure 1B,C, respectively, with the Brb-loaded micelles having a slightly larger size (27.3 nm) compared to the empty cationic micelles (22.9 nm) (Table 1). The polydispersity indices of the prepared micelles were pharmaceutically acceptable (<0.3), which indicates a homogenous size distribution of the cationic micellar platform (Table 1). Analogously, the Brb-loaded anionic micelles had a slightly larger size (25.4 nm) compared to the empty anionic micelles (21.7 nm) (Table 1), also with polydispersity indices of less than 0.3 for the anionic micelle variety.

Incorporating the PEGylated vitamin E TPGS was designed to enhance the Brb drug loading due to the hydrophobic fragment in its molecules. Compared to single-component phospholipid polymeric micelles, this increases the core and corona volumes and, consequently, the overall micellar solubilization capacity for Brb and other poorly soluble polycyclic drugs. Hence, it was previously demonstrated that this mMic system attained a ~300% increase in Brb solubilization compared to the free drug in an aqueous solution [14,25,29,30].

The zeta potentials of the prepared empty and Brb-loaded cationic micelles were in the positive range due to the presence of OA in the micelles (Figure 1D,E respectively), with values of 16.8 mV and 18.2 mV, respectively (Table 1), confirming the successful incorporation of OA in the micellar corona. This contrasts against the empty and Brb-loaded anionic micelles, with typically negative zeta potentials of −27.3 mV and −25.9 mV, respectively (Table 1).

The physico-chemical stability of the micelles was evaluated at 90 days post-preparation (Table 1). There was no significant change in the measured particle size of either variety of the Brb-loaded micelles—the anionic or cationic micelles—over this period. On the other hand, there was a slight increase in the particle size of the empty versions of the anionic and cationic micelles. When considering anionic vs. cationic micelle drug formulations, there was no significant difference in drug (Brb) loading at the time of formulation (D_0_). Furthermore, there was no significant difference in Brb encapsulation efficiency measured after 90 days of cold storage (EE ≥ 95% for either formulation). The virtually unchanged particle size and superior EE data in Table 1, post-90 days of fabrication, confirm the pharmaceutical stability of the micellar platforms for at least three months of storage under refrigerated conditions (4–8 °C).

### 2.2. In Vitro Release, Biocompatibility, and Hemocompatibility of Brb Nanoformulations

**Figure 2:**
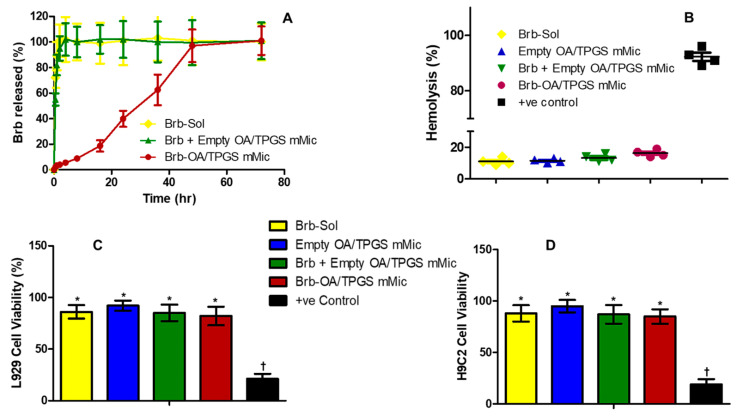
In vitro analysis of cationic vitamin E-TPGS mixed micelles of berberine (Brb). In vitro temporal release profiles of various berberine and cationic micelles formulation (**A**), under sink conditions using 0.05% tween 80 in buffer saline medium, pH 7.4, and incubated for 72 h at 37 °C. (*n* = 5, mean ± SD); Hemolysis test (**B**), after incubation of samples (equivalent to 1.0 µg/mL of Brb) with red blood cells for 3 h at 37 °C (*n* = 3, mean ± SE); in vitro biocompatibility assays, evaluated using murine subcutaneous connective tissue fibroblast (L929) and rat cardiomyocyte (H9C2) cell culture models (panels (**C**) and (**D**), respectively), post-48 h incubation with samples at Brb-equivalent concentration of 10 μM (*n* = 4, mean ± SE, values denoted with unlike symbols (*, †) are statistically different, *p* ≤ 0.05).

The in vitro temporal Brb release from the cationic micelles under pseudo-sink conditions in phosphate buffer saline, at pH 7.4, was distinctly slower compared to both drug controls, the Brb solution, and the admixture of the Brb solution and empty cationic micelles. Figure 2A demonstrates that only 19.4 ± 3.5% of the micelle-loaded Brb was released within the first 18 h, compared to the instantaneous, almost complete Brb presence in the release medium. In addition, the cationic micelle formulation exhibited a marked controlled release profile until 48 h, when nearly 100% of the Brb was released in vitro. (Figure 2A).

Figure 2B, demonstrates the in vitro hemolysis assay that was carried out using a Brb concentration of 1.0 µg/mL (relevant to an IV bolus administration scenario of 1–2 mg/Kg) [14,23]. Compared to RBCs suspended in distilled water (positive control) that showed excessive hemolytic impact (92.5 ± 4.6%), all test solutions resulted in significantly far less hemolysis (*p* ≤ 0.001), with the Brb cationic micelles showing slightly higher hemolysis (17.8 ± 3.1%) compared to the Brb solution, empty cationic micelle platform, and extemporaneous mixture of Brb with empty cationic micelles (14.8 ± 2.9%, 13.7 ± 2.4%, and 15.6 ± 2.5%, respectively, *p* ≤ 0.05). (Figure 2B).

The in vitro biocompatibility assay was evaluated using the L929 and H9C2 cell lines, resulting in similar patterns in both cell lines (Figure 2C,D). All treatments, apart from the CCCP positive-control-treated cells, retained very good viabilities, generally above 85%, without any statistically significant difference amongst them. The apoptotic CCCP treatment (serving as a positive control) reduced the viable cells to below 18.5% overall across both mammalian cell cultures, revealing the remarkable cell viability profiles for all test samples (with or without Brb, *p* ≤ 0.001). Furthermore, Brb, at a test concentration of 1.0 µg/mL, whether free in solution, in a mixture with empty cationic micelles, or encapsulated in the cationic micelles, demonstrated an insignificant change in cell viability in comparison to the empty cationic micelle platform.

### 2.3. Cationic Brb–Mixed Micelles Help Protect against Anthracycline-Induced Cardiovascular Cell Death at a Cytotoxic Level of DOX

**Figure 3:**
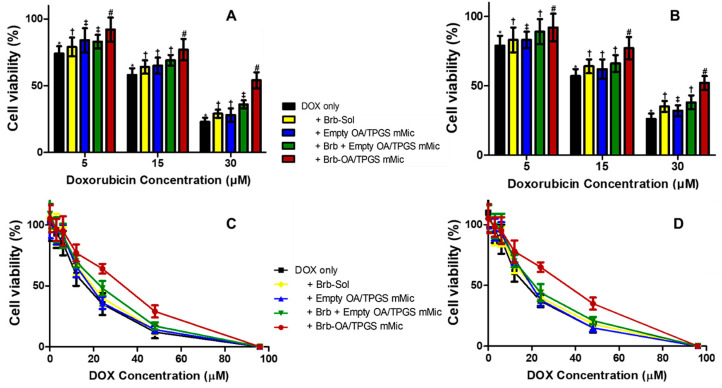
Concentration-dependent recovery of treated rat cardiomyocyte (H9C2, left panels) and mouse aortic medial smooth muscle (A10, right panels) cell models, post-elevated DOX exposure. Microplate-based CellTiter Glo^®^ cytotoxicity assay in cultured H9C2 (**A**) and A10 (**B**) cells, which were treated with 1.0 µg/mL of Brb-Sol, and equivalent concentrations of empty and Brb-loaded micellar formulations, immediately co-exposed for 8 h to acute DOX dose of 5 µM, 15 µM, and 30 µM, to assess mainly DOX-induced apoptotic cell death; overall cell viability profile assessed after 24 h via Cytotox-ONE cell membrane integrity assay of cultured H9C2 (**C**) and A10 (**D**) cells, which were treated with 1.0 µg/mL of Brb-Sol, and equivalent concentrations of empty and Brb-loaded micellar formulations, immediately co-exposed to extended DOX challenge of doses of 0-to-96 µM, all incubated at 37 °C, 5% CO_2_ conditions. (*n* = 4–5, mean ± SE, values denoted with unlike symbols (*, †, ‡, #) are statistically different, *p* ≤ 0.05).

The protective effect of the Brb-loaded cationic micelles against exposure to increasing cytotoxic concentrations of DOX was evaluated in both H9C2 and A10 cell lines. Cell viability was first assessed using a CellTiter Glo^®^ viability assay, Promega (Madison, WI, USA), which determines the number of viable cells based on their metabolic activity via ATP quantitation, and is indicative of apoptotic cell fraction, generally measured post 6–10 h of exposure to DOX (Figure 3A,B). In the H9C2 cell culture, the DOX-only treatment resulted in a decrease in cell viability from 74.8 ± 5.5% to 58.3 ± 4.9% and down to 23.4 ± 3.2% upon exposure to DOX concentrations of 5, 15, and 30 µM, respectively. A similar pattern was observed for the A10 cells. Co-treatment with the Brb solution (1.0 µg/mL) resulted in a minimal yet statistically significant improvement in cell viability in both test cell lines, which was also similarly observed following the treatment with empty cationic micelles, and a mixture of Brb and cationic micelles resulted in slight improvement in cell viability (*p* ≥ 0.05). On the other hand, the most significant improvement in cell viability was observed after treatment with the Brb-loaded cationic micelles (*p* ≤ 0.01), reaching up to 92.5 ± 7.4%, 77.2 ± 6.8%, and 54.5 ± 6.1% in the H9C2 cell lines, and 93.4 ± 8.6%, 76.5 ± 7.7%, and 53.1 ± 5.8% in the A10 cells (corresponding to DOX concentrations of 5, 15, and 30 µM, respectively).

To further confirm the cardioprotective effect of the Brb-loaded cationic micelles against the cytotoxic impact of DOX exposure, the cytotoxicity profiles were also evaluated by nonviable cell signal, using the membrane integrity assay via measuring the release of LDH (Figure 3C,D). In both cell line models, the DOX solution exhibited the strongest cytotoxicity profile overall, with less than 15% of cells being viable at 48 µM of drug. In parallel with the corresponding data in Figure 3A,B, the co-treatments with the Brb solution, empty cationic micelles, and Brb mixture with cationic micelles resulted in a marginal improvement in cell viability against the effects of the DOX challenge over the same concentration range, At the same time, the co-treatment with Brb-loaded cationic micelles resulted in the highest cell viability profile overall, with the most significant improvements being observed at elevated cytotoxic DOX concentrations (12 µM < DOX < 96 µM) at each concentration of DOX, consistently in both cell lines compared to the other controls [27,29].

Table 2 demonstrates the cardioprotective effect of the Brb-loaded cationic micelles against a DOX challenge through IC50 values, calculated based on the in vitro data from both cardiac cell line cultures. A significant increase in the calculated IC50 values was observed in the H9C2 cardiomyocytes (≥1.65 times, *p* ≤ 0.05), from 19.6 µM when treated with DOX only to 31.8 µM in the case of the Brb-loaded cationic micelles co-treatment At the same time, the co-treatments with the Brb solution, empty cationic micelles, and Brb mixture with empty cationic micelles resulted in less substantial improvements in the IC50 values of the DOX-treated H9C2 cells with (<1.2 folds overall). A similar pattern was observed in the A10 cells, where the IC50 values for the cells treated with DOX only vs. cotreatment with Brb-loaded cationic micelles were approx. 18.2 µM and 29.7 µM, respectively (i.e., significantly increased by >1.7 folds, *p* ≤ 0.05), while the other cotreatments resulted in a more modest increase in IC50 values, averaging merely between 19.5 µM and 21.5 µM.

### 2.4. Recovery of Mitochondrial Membrane Integrity and Function by Cationic Brb–Mixed Micelles following DOX-Induced Damage

**Figure 4:**
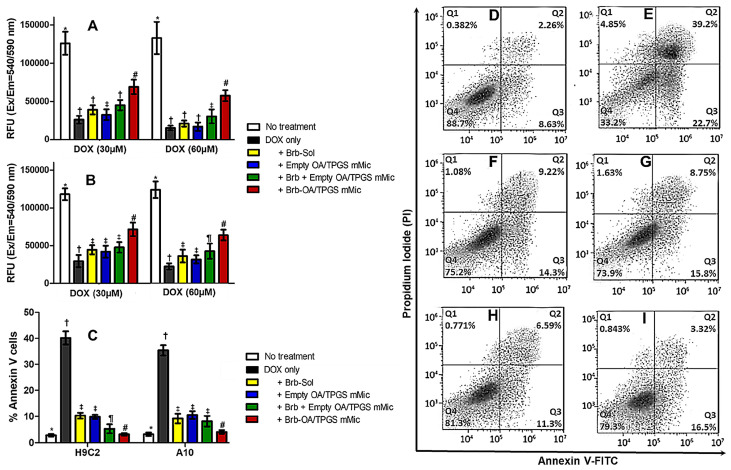
Evaluation of mitochondrial protection against DOX-induced cardiovascular cell apoptosis after micellar berberine treatments. Microplate-based MitoOrange Dye fluorescence assay using cultured H9C2 (**A**) and A10 (**B**) cells, which were exposed to acute DOX challenge at two dose levels of 30 µM and 60 µM, co-treated with 1.0 µg/mL of Brb-Sol, and equivalent concentrations of empty and Brb-loaded micellar formulations, and then fluorescence intensity was measured after 4 h; cellular apoptosis assayed using Annexin V-FITC (panels (**C**–**I**)), following 6 h co-incubation with 30 µM DOX challenge, along with the corresponding 1.0 µg/mL of Brb-treatments (as free Sol, admixtured or encapsulated with OA/TPGS mMic) and equivalent empty OA/TPGS mMic control. Apoptotic cells were detected by flow cytometry. The mean fluorescence intensity of Annexin V/PI staining in both H9C2 myocytes and A10 SMCs was calculated (**C**), following quantitative FACS analysis, as demonstrated in H9C2 myocytes (panels (**D**–**I**)). All cells were cultured in standard 37 °C, 5% CO_2_ conditions. (*n* = 4, mean ± SD values denoted with unlike symbols (*, #, †, ‡, ¶) are statistically different, *p* ≤ 0.05).

The specific ability of the cationic Brb–micelles to restore mitochondrial membrane integrity and function following a DOX challenge was directly quantified by fluorescent staining in cultured H9C2 and A10 cells, where the MitoOrange dye accumulation depended upon the mitochondrial membrane potential and integrity (Figure 4A,B). In both DOX-treated cell lines, the evident recovery of mitochondrial membrane function due to co-treatment with the Brb-loaded cationic micelles was significantly higher than that observed with the co-treatments with the Brb solution (*p* ≤ 0.05), empty micelles solution (*p* ≤ 0.01), and admixture of the Brb solution and cationic micelles solution (*p* ≤ 0.01), indicating the superior mitochondrial membrane stabilization efficacy of Brb loaded cationic micelles. This mitochondria-specific cardioprotective effect was detected with both DOX acute treatment doses of 30 µM and 60 µM.

DOX-induced apoptosis was evaluated via Annexin V/PI staining in both H9C2 myocytes and A10 smooth muscle cells following 6 h of co-incubation with 30 µM of DOX, along with the corresponding 1.0 µg/mL of Brb treatments, as well as the empty cationic micelles (Figure 4C). The acute treatment with DOX only resulted in the highest percentage of apoptotic cells. In contrast, co-treatment with the Brb solution, empty cationic micelles, Brb mixture with cationic micelles, and Brb cationic micelles demonstrated a marked and significant decrease in the percentage of apoptotic cells (*p* ≤ 0.001), with the Brb-loaded cationic micelles showing the lowest cellular apoptosis (down to 13.7 ± 2.4%, and 15.6 ± 2.5%, in H9C2 and A10 cells, respectively) among all DOX co-treatments.

Figure 4D–I show an FACS analysis with Annexin V FITC/PI in H9C2 cardiomyocytes, where healthy, untreated cells were localized in Q4 (Figure 4D). In contrast, the DOX-only-treated cells (Figure 4E) had only 33.2% of cells in Q4, leaving a large percentage of dying cells undergoing apoptosis and/or necrosis—up to 39.2% (in Q2). As seen in Figure 4F–I, co-treatment with either the Brb solution, empty cationic micelles, simple Brb mixture with empty cationic micelles, and Brb-loaded cationic micelles resulted in restoring the healthy cell population (quantified in Q4) to values between 73.9% and 81.3%. Most notably, the Brb-loaded cationic mixed micelles co-treatment with DOX displayed the lowest percentage of H9C2 cells in late apoptosis (down to 3.32% in Q2) compared to all other co-treatments.

### 2.5. Significant Relative Reduction in Apoptotic Caspase-9 and Elevation of Antiapoptotic Bcl-2 Mediates Mitohormetic Cationic Brb–Mixed Micelles Protection against DOX Cardiotoxicity

**Figure 5:**
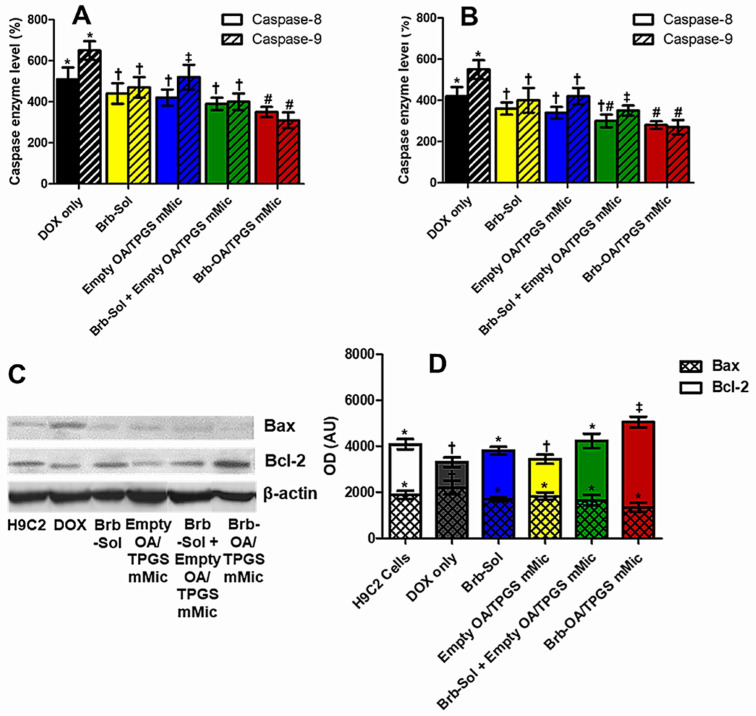
Mitohormetic effects of Brb-mMic activate mitochondrial stress protection against DOX-induced mitochondrial apoptosis in model cardiac cells. Colorimetric microplate Caspase-8 and Caspase-9 activation assays in model H9C2 myocytes (**A**) and A10 SMCs (**B**), where cardiac cells were exposed to acute DOX dose of 15 µM and co-treated with 1.0 µg/mL of Brb-Sol and equivalent concentrations of empty and Brb-loaded micellar formulations, for 4 h at 37 °C in 5% CO_2_ conditions. After washing treated cells, they were normalized by protein content, then incubated with either Caspase-8 or Caspase-9 substrate solution, in reaction buffers according to manufacturer’s instructions; the sample color absorbance values were measured and analyzed relative (%) to the corresponding untreated cell control; Western blot analysis for the relative accumulation of proapoptotic Bax protein and antiapoptotic Bcl-2 protein markers of mitochondrial membrane integrity in rat H9C2 cardiomyocytes treated with acute 15 µM DOX challenge, along with Brb-loaded mMic and blank mMic vehicle control, or un-encapsulated drug controls (either free Brb-solution or a simple mix of Brb-solution + empty mMic), at concentration equivalent to 1.0 µg/mL of Brb for 2 h, respectively. A total of 20 µg of protein extracts was loaded per well for H9C2 cells, and β-actin served as a loading control. Nitrocellulose membrane blots were immunoblotted with either anti-BAX or anti-Bcl-2 monoclonal antibodies, as well as an anti-β-actin monoclonal antibody, and finally visualized (**C**), followed by quantification of intensities of the protein bands using ImageJ Gel analysis (ver. 1.53e), comparing Bcl-2 vs. Bax activation (**D**). (*n* = 3–4, mean ± SD values denoted with unlike symbols (*, †, ‡, #) are statistically different, *p* ≤ 0.05).

DOX exposure at a concentration of 15 µM for 4 h resulted in highly elevated levels of caspase 8 and caspase 9 in H9C2 myocytes and A10 SMCs (Figure 5A,B, respectively), compared to their corresponding basal levels in untreated cells (>400–600% overall), indicating a marked induction of apoptosis. The co-treatments with the Brb solution, empty cationic micelles, Brb mixed with empty cationic micelles, or Brb-loaded cationic micelles (at 1.0 µg/mL of Brb or equivalents) were all associated with significantly lower levels of both caspases 8 and 9, as seen in both model cell lines (*p* ≤ 0.05–0.01). The data also revealed that the mitochondria-avid cationic mixed micelles loaded with Brb (Brb-OA/TPGS mMic) did not demonstrate the lowest expression levels of caspases 8 (aver. 354% and 311% in H9C2 and A10, respectively) and 9 (aver. 282% and 276% in H9C2 and A10, respectively) in both test cell lines, but their associated caspase 8 and 9 marker enzymes were significantly lower than those induced by the closest DOX–cotreatment control, the extemporaneous mixture of Brb-Sol + Empty OA/TPGS mMic (*p* ≤ 0.05).

To mechanistically elucidate the underlying mitochondrial membrane stabilization effect of the Brb-loaded cationic micelles as a DOX co-treatment in H9C2 cardiomyocytes, the levels of mitochondrial pro- and anti-apoptotic signaling proteins, Bax and Bcl-2, respectively, were evaluated. Western blotting was carried out to analyze these protein markers qualitatively (Figure 5C) and quantitatively (Figure 5D).

As shown in Figure 5C, the apparent positive Bax protein band in the cells exposed to the DOX-only treatment indicates a distinctive increase in Bax expression in this sample, compared to all DOX–co-treatment samples, along with the untreated H9C2 cell control. At the same time, visually, there was an almost invisible Bax expression band in the case of the Brb-loaded cationic micelles. Conversely, the cotreatment with Brb-loaded cationic micelles resulted in a significant increase in the expression of Bcl-2 in the DOX-challenged H9C2 cardiomyocytes, evident through its corresponding dense protein band. At the same time, the one developed by the DOX-only treated cells paled in comparison. Further quantification of the protein bands’ intensities was carried out (Figure 5D) for both intrinsic apoptosis signaling proteins, Bax and Bcl-2. Cardiomyocytes exposed to DOX only or in combination with the empty cationic micelle cotreatment showed the highest Bax/Bcl-2 ratios overall, indicating a significant mitochondrial induction of apoptosis in these samples. While the remaining Brb-containing cotreatments resulted in significantly lower Bax/Bcl-2 ratios, the cotreatment of DOX-challenged cells with the prototype Brb-loaded cationic micelles proved to have the lowest Bax/Bcl-2 ratio (*p* ≤ 0.05), thus denoting the least intrinsic induction of apoptosis, even when compared to the closest co-treatment sample, the simple mixture of Brb +empty cationic micelles.

## 3. Discussion

**Figure 6:**
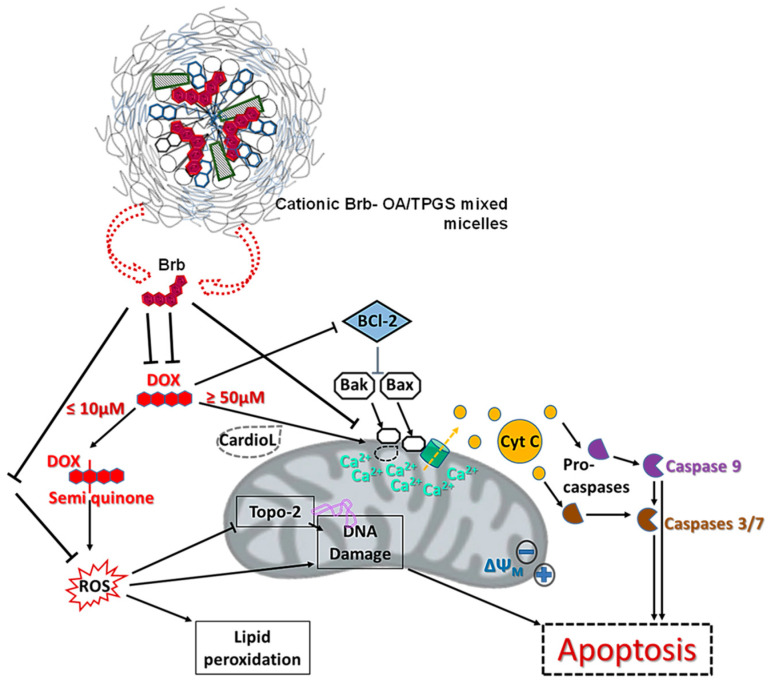
Schematic illustration of putative mito-nuclear mechanisms underlying cardioprotective effects of cationic Brb-mixed micelle treatment against doxorubicin-induced cardiotoxicities.

Doxorubicin is an anthracycline drug widely used to treat various types of solid and hematological cancers, with a severe cumulative risk of cardiomyopathy, primarily through the direct damage of mitochondrial membrane structural integrity, proteins, and overall function. Several pathways have been identified in causing DOX-associated cardiac toxicity (Figure 6).

Generally, DOX undergoes redox cycling in cardiac cells, forming reactive oxygen species (ROS), which can damage many cellular components, including DNA and lipids (REF: Doxorubicin Cardiomyopathy). Following systemic DOX regimens, intracellular iron is substantially increased due to its ability to chelate iron. This further exacerbates ROS-mediated cellular damage overall, yet most notably in the mitochondria [2,4,5]. Chemotherapy doses of DOX also precipitate nuclear and mitochondrial DNA damages, which activate apoptosis in cardiomyocytes, as evidenced by the shift in the balance between anti-apoptotic proteins (e.g., Bcl-2) and pro-apoptotic proteins (e.g., Bax), a crucial step in determining cardiomyocyte fate. Consequently, the increased relative expression of Bax/Bcl-2, as well as the DOX-induced oxidative stress, leads to the release of cytochrome c (Cyt C) into the cytoplasm, where Cyt C participates in the formation of the apoptosome complex, which activates caspase 9—an initiator caspase in the intrinsic or mitochondrial pathway of apoptosis. Activated caspase 9 then activates downstream effector caspases, such as caspases 3/7, which execute the apoptotic death pathway. Additional mechanisms of DOX toxicity in cardiomyocytes involve mitochondrial calcium overload, which results in activating the mitochondrial permeability transition pore (mPTPs), leading to the loss of mitochondrial membrane potential, mitochondrial swelling, and mitochondrial outer membrane rupture, and subsequent Cyt C release [2,4,5].

Accumulated preclinical reports have demonstrated the broad pharmacological activities of Brb, mediated through the drug’s effect on diverse cellular targets and signaling processes, which mainly involve mitochondria as a key effector and relay that supports healthy cell function and maintains cell homeostasis. Yet, realizing the clinical benefits of Brb in stabilizing and restoring mitochondrial function and communications following toxic and pathological myocardial injury, for instance, has been pharmaceutically hampered due to its challenging physicochemical properties and poor systemic bioavailability.

Based on earlier work, our data illustrated the superior pharmaceutical and pharmacological benefits of utilizing PEGylated mMic as a model drug delivery system (DDS) for berberine, thanks to the inclusion of PEGylated vitamin E TPGS, to improve its intracellular accumulation in cardiac tissues and cells rich in mitochondria [14,24,28,30]. The rationale for using cationic PEG-PE/TPGS mMic as DDS for Brb (class III drug) is to enhance their specific interaction with the exceedingly negatively charged mitochondrial membranes, following better internalization of the cationic mMic inside the cells, supported by the TPGS-containing micellar corona [14,29,30] Hence, an overall positive surface charge was achieved by including OA in the mMic structure. At the same time, PEG-PE provided the necessary stealth properties, ensuring micellar stability in the circulation against opsonization for systemic administration [14,24,30,31]. 

The intravenous administration of nanomedicine requires an assessment of its physical and chemical stability during storage and confirmation of its biocompatibility, safety, and pharmaceutical release properties, as expected for this prototype mMic dosage form of Brb. The Brb-loaded cationic micelles did not show any significant difference in particle size or drug loading between measurements at the time of preparation up to 90 days post-storage, which confirms their remarkable pharmaceutical stability. In addition, the produced average size (26.9–27.3 nm) is suitable for the intravenous delivery of the micelles and, most importantly, is quite beneficial for accumulation in both highly and poorly permeable tissues and tumors, based on different reports [24,28,31].

The CMC value of any micelle-forming formulation influences both its in vitro and in vivo stabilities. The very low CMC values for the prototype mMic carriers, composed of PEG_2000_-DSPE and TPGS in a 3:1 molar ratio (previously determined ~2.2 × 10^−5^ M), underlie the several pharmaceutical advantages of this platform, such as its small size (≤50 nm), good solubilization efficacy, low toxicity, controlled drug release, and extremely high physicochemical stability [15,25,29,30]. The stability of the mMic system is evident through its long-term physical stability under standard pharmaceutical refrigerated storage conditions and RT, as reported previously [15,25,30]. Such superior stability, even at 37 °C, underscores the delayed release of Brb from the prototype cationic micelle platform after 12–18 h. Along with the absence of any burst effect (Figure 2A), these in vitro properties confer great pharmaceutical and pharmacokinetic advantages, as they allow for the biodistribution and sufficient accumulation of the drug-loaded micelles within cells before the release of Brb [14,28,32]. Furthermore, the biological safety of the prototype Brb-loaded cationic micelles was evident through the high 80% cell viability observed in L929 and H9C2 cells, as well as the minimal hemolytic effect, as compared to saline-treated sample controls (Figure 2C,D) [33]. Since Brb is considered to be a BCS class III drug with limited solubility and extremely low permeability, its formulation into TPGS-mixed micelles would help to enhance its intracellular uptake for better mitochondrial delivery [14,34,35,36]. While TPGS was previously reported to have some degree of mitochondria-induced apoptosis, the current encouraging biocompatibility and hemocompatibility data (Figure 2C,D) indicate the marginal impact of empty cationic TGPS-mixed micelles on normal cell viabilities overall [10,14,31,33]. Moreover, the mitochondrial apoptosis induction assay results, shown in Figure 4A,B, confirm that, in the context of a DOX challenge, the empty cationic TPGS-mixed micelles did not result in any appreciable adverse effect on mitochondrial viability, in comparison to the native Brb solution treatment.

The presented in vitro cytotoxicity data, following 24 h of an acute DOX challenge, clearly demonstrated the strong cardioprotective effect of Brb co-delivery via the cationic mixed-micelle nanoformulation in both cardiovascular cell models (Figure 3A–D). Pharmacologically, the mMic combinational delivery of Brb proved to be about 1.5–1.6 times more effective than the Brb solution (based on reported IC50 values, Table 2) in mitigating the toxic effect of intense DOX treatments. Even at extremely elevated DOX dosing of ≥50 µM, restoring normal cell viability after co-treatment with the prototype cationic Brb-mMic was significantly superior to all other Brb treatments.

Congruent to various reports, in our study, the direct toxicity of DOX on cardiac mitochondria was evident through the diminished fluorescence of stained mitochondrial membranes, observed in cardiac and vascular cells treated with DOX solution only (Figure 4A,B), indicating a loss of mitochondrial membrane potential. The apoptotic effect was confirmed further by FACS analysis, showing an abundance of cells treated with DOX only in Q2 and Q3, revealing the early and late apoptosis of model cardiac cells. The increased levels of marker caspase enzymes, 9 > 8, (Figure 5A,B), combined with the substantial ratio of Bax/Bcl-2 elevated levels following acute DOX-only exposure (10–15 µM), reaffirms the direct apoptotic induction of the intrinsic mitochondrial pathway by DOX as the primary cardiotoxic mechanism.

At the same time, the enhanced mitohormetic role of the Brb-loaded cationic micelles in protecting cardiac cells from the apoptotic consequences of the DOX challenge was mechanistically confirmed through the preservation of mitochondrial membrane potential, evident in the increased healthy mitochondrial staining with MitoOrange when compared to all other co-treatments (Figure 4A,B), as well as in the reduced fraction of DOX-treated cardiomyocytes that were in early or late apoptotic stages (Figure 4C–I). This can be attributed to the improved mitochondria stabilization effects of Brb at lower doses when delivered via our prototype mixed micelle formulation, all thanks to the added cationic property imparted by OA, combined with the TPGS-enhanced permeability and intracellular uptake, thus collectively mediating an effective mitochondria-tropic delivery of Brb, similar to earlier data reports [29,36,37].

Moreover, the observed markedly significant reduction in the ratio of Bax/Bcl2 elevated levels in the cells exposed to a DOX challenge and co-treated with Brb-loaded cationic mixed micelle indicates the pivotal role of mitochondrial selectivity in enhancing the mitohormetic effects of Brb to mediate cardiovascular cellular resilience.

## 4. Materials and Methods

### 4.1. Materials and Cell Lines

Berberine Hydrochloride (Brb HCl) and cationic oleyl amine lipid were purchased from Sigma-Aldrich (St. Louis, MO, USA). 1,2-Distearoyl-sn-glycero-3-phosphoethanolamine-N-[methoxy(polyethyleneglycol)-2000] (mPEG2000-DSPE or PEG-PE) and ovine wool cholesterol were purchased from Avanti Polar Lipids, Inc. (Alabaster, AL, USA) and used without further purification. Speziol^®^ TPGS-Pharma (NF-grade Vitamin E polyethylene glycol succinate, TPGS) was received as a gift from Cognis (Cincinnati, OH, USA). All other reagents and components of buffer solutions were analytical-grade preparations. An orange mitochondrial membrane potential assay kit, colorimetric Caspase-8 and fluorometric Caspase-9 Assay Kits (microplate, Abcam, Cambridge, MA, USA), Trypsin/EDTA, penicillin/streptomycin, and fetal bovine serum were obtained from Fisher Scientific (Waltham, MA, USA), while the CellTiter Glo^®^ 2.0 cell viability assay kit, Cytotox-ONE™ membrane integrity assay, and Orange Mitochondrial Membrane Potential Assay Kit (microplate) were purchased from Promega (Madison, WI, USA) and Abcam plc. (Cambridge, MA, USA), respectively. Both 6- and 12-well tissue culture-treated microplate plates and black 96-well tissue culture plates were purchased from BD-biosciences (San Jose, CA, USA). Rat Cardiomyocytes (H9C2), aortic medial smooth muscle cells (A10), and murine subcutaneous connective tissue fibroblasts (L929), along with Dulbecco’s Modified Eagle’s Medium (DMEM) and Eagle’s Medium (EMEM), were purchased from American Type Culture Collections (Manassas, VA, USA). Milli-Q (MQ) water was utilized for all preparations.

### 4.2. Formulation of Berberine–Mixed Micelles

Original anionic mixed micelles (mMic) were prepared using a fixed molar ratio (1:3) mixture of vitamin E TPGS and PEG2000-DSPE, which was then stirred with Brb HCl previously dissolved in warmed ethanol (200 proof) at a 2 mg/mL concentration, followed by solvent evaporation. Then, HEPES-buffered saline (HBS) solution, at pH 7.4, was added to the formed lipid film, leading to a clear micelle (Mic) solution after 10 min of 800 rpm vortex mixing. Cationic mMic were similarly prepared by adding 5 M% of the cationic moiety, oleyl anime (OA), into the starting 3:1 molar mixture of vitamin E TPGS and PEG2000-DSPE, prior to stirring in an ethanolic solution of Brb HCl, followed by final solvent evaporation to form the 5%OA/3:1 PEG-DSPE: TPGS lipid film, as described. All resulting mMic dispersions were filtered twice with 0.22 μm pore size polycarbonate membrane filters to remove any non-incorporated Brb and sterilize the mMic formulations. The filtrates were stored in vials under argon until further use [25,37]. Alternatively, the filtered mic samples could be freeze-dried by storing aliquots of the filtrates in 5 mL glass vials, following freezing in liquid nitrogen and vacuum-drying (FreeZone 4.5 benchtop lyophilizer, Labconco, Kansas City, MO, USA, *p* < 200 × 10^−3^ bar, condenser temperature ≤−50 °C). Following lyophilization, the samples were sealed under argon and stored at 4 °C until use. Table 1 lists some of the properties of the produced cationic (5%OA-containing) vs. original anionic 3:1 PEG-DSPE: TPGS mMic formulations [14,25,29].

### 4.3. Physicochemical Characterization of Brb-Containing Cationic Mixed Micelles

#### 4.3.1. Particle Size Analysis

The mMic formulations were diluted with de-ionized distilled water before analysis, and the numbered average particle hydrodynamic diameter and the polydispersity index (PDI) were determined using the dynamic light scattering technique, via Malvern Zetasizer Nano-ZS (Malvern Instruments Inc., Westborough, MA, USA) [14,37].

#### 4.3.2. Zeta Potential (ζ) Measurements

All formulation samples were diluted with de-ionized distilled water, at pH 6.8, placed in the electrophoretic cell of the Malvern Zetasizer Nano-ZS (Nano ZS, Malvern Instruments Inc., Westborough, MA, USA), and the average surface charge was determined [25,37].

### 4.4. Encapsulation Efficiency (EE%) Determination

The amount of the drug, Brb, encapsulated in the cationic mixed micelles (Brb-OA/TPGS mMic) was determined using a modified reversed-phase HPLC method (immediately, D_0_, and then after 90-days of cold storage at 4–8 °C, D_90_). Briefly, a clear aqueous Brb-OA/TPGS mMic dispersion was dissolved in methanol, then, after dilution in the mobile phase, a sample volume of 20 μL through an ACE PFP C-18 column (4.6 × 250 mm, 4 µm packing vol.). The mixture of acetonitrile and 0.05 mol./L NaH_2_PO_4_ (final pH 2.5, adjusted by phosphoric acid), in the ratio of 30:70 *v*/*v*, was utilized as the mobile phase after filtration and degassing. The flow rate was 1.0 mL/minute and U.V. detection was performed at λ_max_ = 346 nm, while the column temperature was maintained at 27 ± 1 °C. The retention time of Brb was about 7 min. The encapsulation efficiency (EE%) of Brb in the OA/TPGS mMic samples was calculated based on standard curves constructed in the range of 4–200 μg/mL (r^2^ = 0.998, *n* = 3), which were validated for linearity, precision, and accuracy [14,36].

### 4.5. Physical Stability of Brb–Micelles

The Brb-loaded mixed micelle formulations (either anionic 3:1 PEG-DSPE: TPGS mMic or cationic 5%OA/3:1 PEG-DSPE: TPGS mMic) were monitored over 3 months of storage in refrigerated conditions (4–8 °C) for time-dependent changes in the physical characteristics (drug precipitation, change in micelle size, and surface charge post-90 days, D_90_) of the formulations. The chemical stability of the Brb in micelles was evaluated by the HPLC, as described above [14,37].

### 4.6. In Vitro Release of Brb from Micelles at “Sink” Conditions

The dialysis bag method was utilized to study the time-dependent release of Brb from the cationic OA/TPGS mMic nanocarriers to non-encapsulated drug. Drug-loaded samples (0.25 mL of approx. 3 mM of Brb) were placed in Spectra/Por^®^ dialysis cellulose ester (CE) membranes (MWCO = 25 KDa, Spectrum Laboratories Inc., Rancho Dominguez, CA, USA) after soaking overnight in release medium. Closed dialysis bags were submerged into 200 mL of test external release medium of phosphate-buffered saline solution (PBS, pH 7.4), containing 0.05% of tween 80, to maintain pseudo-sink conditions in a limited volume, for up to 72 h of incubation with continuous stirring at 200 rpm speed. At specific time intervals, 0.5 mL samples of each release medium were withdrawn and replaced with an equal volume of fresh medium; then, the samples were filtered through 0.22 µm syringe membrane filters. The Brb content in the samples was finally determined by HPLC, as described above [14,25,29].

### 4.7. In Vitro Hemolysis Assay

According to a previously reported assay, hemolysis studies were carried out for the empty cationic OA/TPGS mMic vehicle, along with different Brb-containing samples [27]. The release of hemoglobin from the erythrocytes was used for toxicity measurements of these carriers. Briefly, defibrinated rabbit blood (Thermo Scientific, Waltham, MA, USA) was diluted ten times with PBS and centrifuged at 2000 rpm for 15 min. The supernatant was decanted, and the precipitate was rinsed three times with PBS, followed by centrifugation at 2000 rpm for 15 min. The concentration of the resulting blood cells was adjusted to 2% (*v*/*v*). In a physiologically relevant dilution scenario of IV bolus administration (1.0 µg/mL of Brb-equivalent concentration), 50 µL of test samples was mixed with 500 μL of blood cells, and the resulting suspensions were incubated at 37 °C for 3 h. The samples were then centrifuged at 2000 rpm for 15 min. The absorbance of the supernatant was measured at 540 nm to determine the amount of hemoglobin released. Zero hemolysis and 100% hemolysis were red blood cells suspended in physiological saline −ve control) and distilled water (+ve control), respectively.

The percentage of hemolysis was determined by the following Equation (1):

(1) $$Hemolysis\;\left(\%\right)=\frac{Ats-A0}{\left(A100-A0\right)}\times 100$$

where *Ats* is the absorbance of the test sample, *A*100 is the absorbance of completely lysed red blood cells in distilled water, and *A*0 is the absorbance of zero hemolysis [34].

### 4.8. In Vitro Cell Culture

Rat cardiomyocytes (H9C2), aortic medial smooth muscle cells, and SMCs (A10) were grown in a complete Dulbecco-modified MEM (DMEM) culture medium. In contrast, subcutaneous connective tissue mouse fibroblasts (L929) were grown in complete Eagle’s MEM (EMEM) culture medium—prepared by adding 10% fetal bovine serum and penicillin (100 U/mL)/streptomycin (100 μg/mL)—in a humidified environment of 37 °C and 5% CO_2_. Cells were seeded at a concentration of 6 × 10^3^–1.5 × 10^4^ cells/cm^2^ and sub-cultured at approximately 70–80% confluency. Cells between passages 6 and 12 were used for experimentation [25,26,37].

### 4.9. In Vitro Biocompatibility Screen

Murine subcutaneous connective tissue fibroblasts (L929) and rat cardiomyocytes (H9C2) were seeded at 10 × 10^3^ cells per well of 96-well plates in triplicate for 24 h. Then, the respective complete culture media were exchanged for serum-free media only (serving as the no-treatment cell culture negative control, −ve control) or containing 10 µM of free Brb dissolved in hydro-alcoholic solution (Brb-Sol), compared with the equivalent concentrations of the empty cationic OA/TPGS mMic vehicle, a simple admixture of the two, and the corresponding Brb-loaded cationic OA/TPGS mMic formulation. CCCP was included to induce apoptosis as a positive (+ve) control for the same period and incubation conditions. Finally, after 48 h of co-incubation, total cell viability was determined using the Cytotox-ONE™ lactate dehydrogenase (LDH) release assay Kit (Promega, Madison, WI, USA) after washing twice with Hank’s Balanced Saline solution, pH 7.4 (HBSS) and reading sample plate fluorescence (λ_Ex_ = 480 nm/λ_Em_ = 530 nm), using the Synergy 2 Biotek fluorescence plate reader (Biotek instruments Co., Winooski, VT, USA), according to the manufacturer’s instructions [29,34].

### 4.10. Cell Viability Assays following Doxorubicin Challenge

Rat cardiomyocytes (H9C2) and aortic medial smooth muscle cells (A10) were seeded at 20 × 10^3^ cells per well in 96-well microplates in five replicates for 24 h. The complete culture media were then exchanged for serum-free media (SFM) containing 1.0 µg/mL of un-encapsulated Brb-Sol, as positive drug control treatments (either alone or admixed with empty OA/TPGS mMic) and drug-equivalent concentrations of Brb-loaded OA/TPGS mMic, plus a blank/empty OA/TPGS mMic vehicle control, all compared with plain SFM serving as a negative control. Immediately afterward, an acute doxorubicin challenge was directly added to the same wells, at 5, 15, and 30 µM of final DOX concentration. Following 8 h of co-incubation at 37 °C in 5% CO_2_, the cell viabilitybased on primarily apoptosis-mediated cell deathwas determined using a CellTiter Glo^®^ assay Kit (Promega, Madison, WI, USA) after washing twice with HBSS and reading sample plate fluorescence (λ_Ex_ = 480 nm/λ_Em_ = 530 nm), using the Synergy 2 Biotek fluorescence plate reader (Biotek Instruments Co., Winooski, VT, USA), according to the manufacturer’s instructions. Similarly, the Cytotox-ONE™ membrane integrity assay was used to measure the LDH release after 24 h of co-incubation with an acute DOX challenge (two-fold serial dilutions, concentration range 0 to 96 µM) and the corresponding 1.0 µg/mL of Brb-treatments (as free Sol, admixtured, or encapsulated with OA/TPGS mMic) and empty OA/TPGS mMic control. The final treated cell viability was determined after washing three times with HBSS and reading sample plate fluorescence (λ_Ex_ = 560 nm/λ_Em_ = 590 nm) using the Synergy 2 Biotek fluorescence plate reader (Biotek Instruments Co., Winooski, VT, USA), according to the manufacturer’s instructions [20,26,37].

### 4.11. Apoptosis Assays

#### 4.11.1. Flow Cytometry

According to the manufacturer’s protocol, apoptosis was measured using Annexin V-FITC green dye of the Apoptotic, Necrotic, and Healthy Cells Quantitation Kit Plus (Biotium, Inc., Fremont, CA, USA). Following 6 h of co-incubation with a 30 µM DOX challenge, along with the corresponding 1.0 µg/mL of Brb treatments (as free Sol, admixtured, or encapsulated with OA/TPGS mMic) and equivalent empty OA/TPGS mMic control, cells (seeded at 1.5 × 10^6^), were then incubated with 5 µL of Annexin V-FITC and 5 µL of PI at room temperature for 15 min in the dark. Apoptotic cells were detected by flow cytometry. The mean fluorescence intensity of Annexin V/PI staining in the myocytes and SMCs was analyzed using the BD FACSCalibur™ (BD Biosciences, Franklin Lakes, NJ, USA) [13].

#### 4.11.2. Orange Mitochondrial Polarization Assay

Approximately 1.0 × 10^6^ cells—from either rat H9C2 cardiomyocytes or A10 aortic SMCs—were grown overnight to attach inside 6-well culture plates. The cells were incubated for 4 h with a 30 µM DOX challenge, along with un-encapsulated drug controls (either free Brb solution or simple admixture of Brb solution + empty OA/TPGS mMic) at a concentration equivalent to 1.0 µg/mL of Brb drug. The cells were then washed, trypsinized, collected, and normalized by their protein content, determined per standard BCA protein assay kit protocol (Thermo Fischer Scientific, Rockford, IL, USA). Afterward, 100 µL of the Orange Mitochondrial Membrane Potential Assay (microplate, Abcam, Cambridge, MA, USA) dye working solution was added to each well containing 100 µL of the sample media. After 30 s of mixing, the plate contents were incubated for 30 min. Finally, the sample plate fluorescence intensity values were measured (λ_ex_/λ_em_: 540 nm/590 nm) after adding Assay Buffer B, using the Synergy 2 Biotek fluorescence plate reader (Biotek Instruments Co., Winooski, VT, USA), according to the manufacturer’s instructions. The orange fluorescence intensity in normal cells increased as the cationic MitoOrange Dye accumulatds in the mitochondria. However, in apoptotic cells, the fluorescence intensity of MitoOrange Dye decreased following the collapse of MMP [26,37].

### 4.12. Mitochondrial Apoptosis Marker Evaluation

#### 4.12.1. Caspase 8 and 9 Activation Assays

Approximately 2.0 × 10^6^ cells—used from each of rat H9C2 cardiomyocytes or A10 aortic SMCs—were grown overnight to attach into 6-well culture plates. The cells were incubated for 4 h with a 15 µM DOX challenge, along with Brb-loaded mMic and blank mMix vehicle control, along with un-encapsulated drug controls (either free Brb-solution or a simple mix of Brb-solution + empty mMic), at a concentration equivalent to 1.0 µg/mL of Brb drug. The cells were then washed, trypsinized, collected, and normalized by their protein content following 30 min of incubation in ice-cold lysis buffer, determined as per the standard BCA protein assay kit protocol (Thermo Fischer Scientific, Rockford, IL, USA). Caspase-8 activity was measured using the colorimetric Caspase-8 Assay Kit (microplate, Abcam, Cambridge, MA, USA), where 50 µL of reaction Buffer/DTT mixture plus 5 µL of LEHD-AFC substrate solution (200 mM final substrate concentration) were added to each well of a 96-well microplate, containing 50 µL of sample media. After 30 s of mixing, the plate contents were incubated at 37 °C for 2 h. Finally, according to the manufacturer’s instructions, the sample color absorbance values were measured (λ_em_ = 400 nm) using a Synergy 2 Biotek fluorescence plate reader (Biotek Instruments Co., Winooski, VT, USA). Analogously, Caspase-9 activity was measured using the fluorometric Caspase-9 Assay Kit (microplate, Abcam, Cambridge, MA, USA), where 50 µL of reaction Buffer/DTT mixture plus 5 µL of IETD-pNA substrate solution e (50 μM final concentration) were added to each well of a 96-well microplate, containing 50 µL of sample media. After 30 s of mixing, the plate contents were incubated for 2 h. Finally, the sample plate fluorescence values were measured (λ_ex_/λ_em_: 400 nm/505 nm) using the Synergy 2 Biotek fluorescence plate reader (Biotek Instruments Co., Winooski, VT, USA), according to the manufacturer’s instructions. The specific caspase-8/-9 activity is reported as the percent (%) induction relative to the untreated control [26,37].

#### 4.12.2. Evaluation of Bax and Bcl-2 Activation by Western Blotting

Rat H9C2 cardiomyocytes and A10 aortic SMCs cells growing in T-25 flasks were treated with a 15 µM DOX challenge, along with Brb-loaded mMic and blank mMix vehicle controls, along with un-encapsulated drug controls (either free Br -solution or a simple mix of Brb solution + empty mMic), at a concentration equivalent to 1.0 µg/mL of Brb drug, for 2 h. Then, the cells were lysed and the total protein was extracted and quantified by a BCA protein assay kit. Subsequently, the protein was separated on a 12% SDS-PAGE gradient gel and then transferred onto the nitrocellulose membrane. Nitrocellulose membrane blots were immunoblotted with either anti-Bax or anti-Bcl-2 monoclonal antibodies, as well as anti-β-actin monoclonal antibody (Santa Cruz Biotechnology Inc., Santa Cruz, CA, USA) overnight at 4 °C, followed by 1 h of incubation at R.T. with a horseradish peroxidase-conjugated secondary antibody [38]. To visualize the protein of interest (i.e., Bax and Bcl-2) along with the internal control (β-actin), the membranes were then incubated for 5 min with an enhanced chemiluminescent substrate, and the exposed film was finally imaged using a Kodak Gel-logic 200 imaging system (Carestream Health, Rochester, NY, USA), followed by quantification of the intensities of the protein bands using ImageJ Gel Analysis software (National Institutes of Health, Bethesda, MD, USA) [38].

### 4.13. Data Analysis

A minimum of triplicates was run for each experiment unless indicated differently. Data are reported as mean ± standard error (SE) unless noted otherwise. Comparisons between two groups were made using the student’s t-test, and for more than two groups, the Kruskal–Wallace test with Tukey’s post-hoc analysis was used to compare results. *p* < 0.05 values are considered to be statistically significant. All statistical analyses were performed using GraphPad Prism^®^ software, ver. 5.0 [15,37].

## 5. Conclusions

The proposed cationic mMic platform, loaded with Brb, demonstrated considerable pharmaceutical properties. In addition, at sub-lethal doses, the cationic Brb-loaded mMic nanoformulation selectively triggers the mitochondria-mediated stress protection of cardiac cells following acute exposures to the anthracycline DOX. This cardio-protective action involves the stabilization of mPTP, activation of antiapoptotic Bcl-2, and blunting of pro-apoptotic Bax levels, thus resulting in a marked reduction in the associated effector caspase marker enzymes and the overall fraction of DOX-treated cardiomyocytes undergoing early or late apoptotic cell death.

Overall, pharmaceutical and pharmacological data suggest a strong potential to develop safe and effective mitohormesis and stress protection treatment using low doses of a Brb-loaded mitochondria-tropic cationic mixed micellar delivery system. Such a mitohormetic nanoformulation of Brb can be explored for systemic administration as a “cardioprotective” adjuvant alongside the primary DOX-containing cancer chemotherapy to help mitigate the dose-dependent cardiotoxic side effects.

## Figures and Tables

**Table 1:** Physico-chemical properties and 90-day (D_0_–D_90_) cold storage stability data of different berberine (Brb) mixed micelle preparations.

Formulation (Lipid Phase, M)	Brb Loading (mg/mL)	Particle Size (nm), D_0_	PDI	Zeta Potential, ζ (mV)	Particle Size (nm), D_90_	Brb Encapsulation Efficiency, D_90_ (EE %)
**3:1 PEG-DSPE:TPGS**	Empty	21.7 ± 1.6 *	0.263	−27.3 ± 1.5 *	26.8 ± 1.4 *	N/A
	1.89 ± 0.2 *	25.4 ± 1.5 ^†^	0.247	−25.9 ± 1.7 *	27.4 ± 2.1 ^†^	96.4 ± 5.7 *
**5%OA/3:1 PEG-DSPE:TPGS**	Empty	22.9 ± 2.1 *	0.274	+16.8 ± 2.3 ^†^	23.2 ± 1.9 ^†^	N/A
	1.94 ± 0.14 *	27.3 ± 1.6 ^‡^	0.255	+18.2 ± 1.1 ^†^	26.9 ± 1.8 ^‡^	94.9 ± 6.8 *

N/A: not applicable, *n* = 3–4, mean ± SD, values denoted with unlike symbols (*, †, ‡) are statistically different, *p* ≤ 0.05.

**Table 2:** Comparison of model cardiac cell viabilities, based on calculated IC50 values, 24 h after acute doxorubicin exposure (0–96 µM), and treatment with berberine solution vs. empty and berberine (Brb)-loaded cationic mixed micelles (at Brb-equivalent conc. = 1.0 µg/mL).

Treatment	IC50 in H9C2 (µM)	IC50 in A10 (µM)
**DOX only**	19.6 ± 3.5 *	18.2 ± 2.9 *
**DOX + Brb-Sol**	23.8 ± 2.8 ^†^	21.5 ± 3.4 ^†^
**DOX + Empty OA/TPGS mMic**	21.2 ± 2.5 ^‡^	19.4 ± 3.1 *
**DOX + Brb + Empty OA/TPGS mMic**	22.4 ± 3.7 ^†^	20.6 ± 3.8 ^†^
**DOX + Brb-OA/TPGS mMic**	31.8 ± 5.1 ^#^	29.7 ± 4.8 ^#^

*n* = 4–5, mean ± SD, values denoted with unlike symbols (*, #, †, ‡) are statistically different, *p* ≤ 0.05.

## Data Availability

The data and contributions presented in the study are included in the article. Further inquiries can be directed to the corresponding author.
